# Impact of follow-up time and analytical approaches to account for reverse causality on the association between physical activity and health outcomes in UK Biobank

**DOI:** 10.1093/ije/dyz212

**Published:** 2019-10-25

**Authors:** Tessa Strain, Katrien Wijndaele, Stephen J Sharp, Paddy C Dempsey, Nick Wareham, Søren Brage

**Affiliations:** 1 MRC Epidemiology Unit, Institute of Metabolic Science, University of Cambridge, Cambridge, UK; 2 Physical Activity & Behavioural Epidemiology Laboratories, Baker Heart and Diabetes Institute, Melbourne, Australia

**Keywords:** Exercise, physical activity, epidemiologic methods, prospective studies, follow-up studies, bias

## Abstract

**Background:**

The advent of very large cohort studies (*n* > 500 000) has given rise to prospective analyses of health outcomes being undertaken after short (<4 years) follow-up periods. However, these studies are potentially at risk of reverse causality bias. We investigated differences in the associations between self-reported physical activity and all-cause and cardiovascular disease (CVD) mortality, and incident CVD, using different follow-up time cut-offs and methods to account for reverse causality bias.

**Methods:**

Data were from *n* = 452 933 UK Biobank participants, aged 38–73 years at baseline. Median available follow-up time was 7 years (for all-cause and CVD mortality) and 6.1 years (for incident CVD). We additionally analysed associations at 1-, 2- and 4-year cut-offs after baseline. We fit up to four models: (1) adjusting for prevalent CVD and cancer, (2) excluding prevalent disease, (3) and (4) Model 2 excluding incident cases in the first 12 and 24 months, respectively.

**Results:**

The strength of associations decreased as follow-up time cut-off increased. For all-cause mortality, Model 1 hazard ratios were 0.73 (0.69–0.78) after 1 year and 0.86 (0.84–0.87) after 7 years. Associations were weaker with increasing control for possible reverse causality. After 7-years follow-up, the hazard ratios were 0.86 (0.84–0.87) and 0.88 (0.86–0.90) for Models 1 and 4, respectively. Associations with CVD outcomes followed similar trends.

**Conclusions:**

As analyses with longer follow-up times and increased control for reverse causality showed weaker associations, there are implications for the decision about when to analyse a cohort study with ongoing data collection, the interpretation of study results and their contribution to meta-analyses.

**Key Messages**

- Analyses with shorter follow-up times showed stronger associations between self-reported physical activity and all-cause mortality, cardiovascular disease mortality and incident cardiovascular disease.
- Different methods to account for reverse causality bias also influenced the strength of the association: least controlled analyses showed stronger associations.
- The magnitude of the differences between approaches varied by outcome.
- This has implications for the decision about when to analyse a cohort study with ongoing data collection, as well as the interpretation of study results and their contribution to meta-analyses.

## Introduction

Many prospective cohort studies have demonstrated prospective associations between lifestyle behaviours and the risk of mortality or morbidity.^1–3^ Typically, follow-up periods have been at least 5 years, and often >10 years, because it was typically necessary to wait for a sufficient number of events to occur before analyses could be undertaken.^4^ With the advent of very large cohort studies such as the Million Women Study (*n* > 1 000 000) and the UK and China Kadoorie Biobanks (both *n* > 500 000), the number of events rarely limits the possibility of early prospective analyses.^5–8^ However, there is little research to indicate if the length of follow-up time after which analyses are undertaken, combined with the analytical choices made to address issues of reverse causality, impacts on the estimated association between behavioural exposures and health outcomes.

In this study, we focus on physical activity behaviour. Previous work in this field has focussed on the potential biases introduced by using very long follow-up periods (>30 years). Longer durations have generally but not consistently attenuated the protective inverse association between physical activity and all-cause mortality,^9–12^ coronary heart disease incidence and mortality.^12–15^ Andersen^9^ attributed this to violation of the constant exposure assumption, i.e. real changes in physical activity behaviour during that time.

For substantially shorter follow-up periods, a major issue of concern is reverse causality, i.e. the potential that underlying (diagnosed or undiagnosed) illness impacts negatively on physical activity, so the observed association between physical activity and health outcomes is more driven by the causal link between the underlying disease state and subsequent outcome events, rather than the physical activity exposure per se.^16^ This will typically lead to an overestimation of the true association between physical activity and health outcomes. Common methods to account for this bias include removing individuals who have prevalent disease, or those who experience the event soon after baseline, who are presumed to represent those with undiagnosed illness at baseline. However, the latter is not always feasible when the study follow-up period is short or the number of events is low.^7^^,^^8^

The aim of this study was to investigate whether the strength of the associations between physical activity and all-cause mortality and cardiovascular disease (CVD) outcomes varies over different follow-up times in the range 1–7 years. A secondary aim was to examine how different approaches to account for reverse causality impact on the strength of the association.

## Methods

### Data source

We used data from UK Biobank, a study involving over 500 000 individuals aged 37–73 years at baseline. Participants undertook an assessment at one of 22 centres across Great Britain between 2006 and 2010. This included a touchscreen self-administered questionnaire and anthropometric measurements.^17^^,^^18^ Consent to link these data to the national death registries and to hospital episode inpatient data was also obtained. Questionnaire and mortality data were downloaded on 31 August 2018, containing information from 502 543 participants after withdrawals. The hospital episode data were downloaded on 9 April 2018.

### Physical activity exposure

The questionnaire covered the frequency and duration of individuals’ participation in four types of discretionary physical activity (home and leisure domains) which are considered to be in the moderate-to-vigorous physical activity intensity range:^19^ (i) walking for pleasure, not as a means of transport, (ii) other exercises, e.g. swimming, cycling, keep-fit, bowling, (iii) strenuous sports, and (iv) heavy do-it-yourself (DIY) home maintenance, e.g. weeding, lawn mowing, carpentry, digging. The response options for frequency were: none, once in the last 4 weeks, 2–3 times in last 4 weeks, once per week, 2–3 times per week, 4–5 times per week, or every day. The response options for duration were: <15 min, 15–30 min, 30–60 min, 1–1½ h, 1½–2 h, 2–3 h or >3 h. Responses were scaled to a total weekly duration using the median values (maximum duration of 3½ h), and truncated at 2000 min (*n* = 544 affected). We excluded those missing both frequency and duration for an activity type (*n* = 19 643) and those who completed the different pilot questionnaire (*n* = 3798). We imputed the frequency or duration from the median of all others reporting participation in that activity type (*n* = 5994) if only one was missing.

### Outcomes

The three outcomes of interest were all-cause mortality, CVD mortality and CVD incidence (fatal and non-fatal). CVD was defined as a primary or secondary diagnosis with International Classification of Disease 10th revision codes I20–25 for ischaemic heart disease and I60–69 for cerebrovascular disease. The censor dates differed for the mortality and hospital episode data for different home nations. Participants attending an assessment centre in England and Wales were followed-up for vital status until 31 January 2016 and those attending one in Scotland until 30 November 2015. For total incident CVD (based on mortality records and hospital episode data), censor dates were 31 March 2015 in England, 31 October 2015 in Scotland and 29 February 2016 in Wales.

Eleven individuals were excluded due to inconsistencies in their mortality data (death date prior to interview, diagnosis but no death date, death date but no diagnosis).

### Covariates

Potential confounders were chosen a priori, based on previous literature. Demographic characteristics included age, sex, ethnicity (white/non-white), highest educational level achieved (degree or above/any other qualification/no qualification) and Townsend indicator of deprivation (a continuous index derived from the respondent’s post code, with higher scores indicating higher deprivation). Lifestyle behaviours included smoking status (never/previous/current); alcohol consumption (never/previous drinker/current drinker); addition of salt to food (never or rarely/sometimes/usually or always); consumption of oily fish (never/less than once per week/at least once per week); fruit and vegetable intake (a score of 0–4 was computed from questions asking the frequency of raw and cooked vegetable, fresh and dried fruit intake; respondents were given 1 point if they reported more than 2 portions of each type); consumption of processed or red meat (average days per week derived from questions on the frequency of processed, beef, lamb and pork intake); leisure screen time (combined total duration of reported TV and computer time during leisure time, categorized to <4 h per day/≥4 h per day in line with current estimates of where the risk of all-cause mortality and CVD mortality increases non-linearly^3^); and usual sleep per 24-h period (<7 h/7–8 h/>8 h). We also derived a five-category variable that combined the responses from questions on employment status (unemployed/in paid or self-employment), walk or cycle to work (yes/no) and heavy manual/physical job (yes/no) because the latter two were only applicable to those in work.

Variables indicating self-reported health status included: current prescription of blood pressure medication (yes/no); current prescription of cholesterol medication (yes/no); diabetes (insulin prescription or self-reported diagnosis/neither); paternal or maternal history of heart attack, angina or stroke (yes to either parent/neither); and paternal or maternal history of cancer (yes to either parent/neither). Body mass index (BMI) was derived from height and weight measured at baseline and categorized into under/normal weight (<25 kg/m^2^), overweight (≥25–<30 kg/m^2^) and obese (≥30 kg/m^2^). Individuals with prevalent CVD were identified from their self-reported previous diagnosis of a heart attack, angina or stroke, or a hospital episode with a previous relevant diagnosis. Individuals with prevalent cancer were also identified via self-report or hospital episode data (International Classification of Disease 10th revision codes C00-C99). Missing data in these covariates led to the exclusion of *n* = 26 104 individuals.

### Statistical analyses

Cox regression with age as the underlying timescale was used to estimate the association between physical activity at baseline and all-cause and CVD mortality, and CVD incidence. We used cubic spline regression to examine the nature of the dose–response relationships and identify a transformation of the physical activity variable which would enable us to include this in the model as a continuous variable (i.e. assuming a log–linear association with the hazard), and hence simplify the presentation of the results from different models (see Supplementary Figure 1, available as Supplementary data at *IJE* online). Based on the findings, the physical activity variable (in min/week) was transformed by adding 1 and taking the natural log. We subsequently standardized this variable by subtracting the sample mean and dividing by its standard deviation.

The median available follow-up time was 6.1 years (for incident CVD) and 7 years (for all-cause and CVD mortality). We also used cut-offs at 1, 2 and 4 years of follow-up after baseline. Where possible within each outcome/follow-up time combination, four models were fitted using different methods to account for reverse causality bias: Model 1 = adjustment for prevalent CVD and cancer; Model 2 = exclusion of individuals with prevalent CVD and cancer; Model 3 = Model 2 plus excluding incident cases that occurred within the first year; Model 4 = Model 2 plus excluding incident cases that occurred within the first 2 years. All models were adjusted for potential confounders.

Proportional hazard assumptions for the exposure and all covariates were assessed using log–log plots for each outcome based on Model 4 with the maximum follow-up time. Assessment centre, ethnicity, alcohol consumption and the combined variable for employment status/active commuting/manual work were accounted for by stratification of the baseline hazard function, rather than as covariates in the linear predictor, because they did not always meet the proportional hazard assumptions.

We quantified the potential degree of regression dilution bias using data from two sub-samples that undertook repeat exposure assessment at one of two follow-up visits; these were conducted a median of 4.4 years (*n* = 18 213) and 7.6 years (*n* = 21 205) after baseline. We also created a further sub-sample of those who undertook the first repeat visit <3 years after baseline (*n* = 2122; minimum follow-up 2 years). We standardized the natural log of the minutes of reported physical activity +1 at these visits to the baseline scale (subtracting the baseline mean and dividing by the baseline standard deviation). The coefficient from a linear regression of the follow-up visit variable on the baseline variable (also transformed and standardized as above) was estimated to indicate the degree of stability in the measured exposure variable over time.

We also performed the models without adjustment for BMI, diabetes diagnosis or insulin prescription, blood pressure medication or cholesterol medication as sensitivity analyses, as these covariates could plausibly be on the causal pathway.

## Results

Sample sizes ranged between 384 615 and 452 993 across the different analyses. Table 1 summarizes the baseline characteristics of the cases and non-cases in the analysis samples used to fit Model 1 for each outcome, with the maximum available follow-up time. Cases for all outcomes were less active, older, of higher education level and more overweight than non-cases. They were also more likely to be unemployed, a current smoker, a previous drinker, report higher leisure screen time, take medication and have a history of disease.

**Table 1.** Baseline participant characteristics from the UK Biobank by health status after a median of 7 years of follow-up^a^

	All-cause mortality				CVD mortality				CVD incidence			
	Cases (*n* = 12 277)		Non-cases (*n* = 440 716)		Cases (*n* = 2643)		Non-cases (*n* = 450 350)		Cases (*n* = 30 146)		Non-cases (*n* = 422 847)	
Minutes of discretionary PA, median (IQR)	101.3	(14.1–262.5)	134.5	(45.0–311.3)	76.9	(1.9–243.8)	133.1	(45.0–309.4)	112.5	(18.8–287.8)	135.0	(45.0–311.3)
Age at baseline interview, *n* (%)												
<55 years	1965	(16.0)	174 751	(39.7)	320	(12.1)	176 396	(39.2)	4544	(15.1)	172 172	(40.7)
55–<60 years	1870	(15.2)	80 756	(18.3)	361	(13.7)	82 265	(18.3)	4696	(15.6)	77 930	(18.4)
60–<65 years	3691	(30.1)	105 406	(23.9)	806	(30.5)	108 291	(24.0)	9366	(31.1)	99 731	(23.6)
≥65 years	4751	(38.7)	79 803	(18.1)	1156	(43.7)	83 398	(18.5)	11 540	(38.3)	73 014	(17.3)
Sex, *n* (%)												
Women	4803	(39.1)	240 473	(54.6)	636	(24.1)	244 640	(54.3)	9935	(33.0)	235 341	(55.7)
Men	7474	(60.9)	200 243	(45.4)	2007	(75.9)	205 710	(45.7)	20 211	(67.0)	187 506	(44.3)
Ethnicity, *n* (%)												
White	11 939	(97.2)	418 938	(95.1)	2544	(96.3)	428 333	(95.1)	28 709	(95.2)	402 168	(95.1)
Non-white	338	(2.8)	21 778	(4.9)	99	(3.7)	22 017	(4.9)	1437	(4.8)	20 679	(4.9)
Highest educational level achieved, *n* (%)												
Degree or above	3668	(29.9)	70 646	(16.0)	958	(36.2)	73356	(16.3)	9268	(30.7)	65046	(15.4)
Any other qualification	5600	(45.6)	221 281	(50.2)	1125	(42.6)	225 756	(50.1)	14 186	(47.1)	212 695	(50.3)
No qualification	3009	(24.5)	148 789	(33.8)	560	(21.2)	151 238	(33.6)	6692	(22.2)	145 106	(34.3)
Townsend indicator of multiple deprivation, median (IQR)	−1.7	(−3.4–1.5)	−2.2	(−3.7–0.4)	−1.0	(−3.0–2.2)	−2.2	(−3.7–0.4)	−1.8	(−3.4–1.4)	−2.2	(−3.7–0.4)
Employment and commuting status, *n* (%)												
Unemployed	8222	(67.0)	179 900	(40.8)	1959	(74.1)	186 163	(41.3)	19 542	(64.8)	168 580	(39.9)
Employed, non-manual work, non-active commute	2858	(23.3)	179 549	(40.7)	496	(18.8)	181 911	(40.4)	7437	(24.7)	174 970	(41.4)
Employed, manual work, non-active commute	523	(4.3)	27 709	(6.3)	106	(4.0)	28 126	(6.2)	1528	(5.1)	26 704	(6.3)
Employed, non-manual work, active commute	566	(4.6)	47 205	(10.7)	63	(2.4)	47 708	(10.6)	1352	(4.5)	46 419	(11.0)
Employed, manual work, active commute	108	(0.9)	6353	(1.4)	19	(0.7)	6442	(1.4)	287	(1.0)	6174	(1.5)
Smoking, *n* (%)												
Never	4633	(37.7)	244 195	(55.4)	818	(30.9)	248 010	(55.1)	12 073	(40.0)	236 755	(56.0)
Previous	5218	(42.5)	152 214	(34.5)	1218	(46.1)	156 214	(34.7)	13 658	(45.3)	143 774	(34.0)
Current	2426	(19.8)	44 307	(10.1)	607	(23.0)	46 126	(10.2)	4415	(14.6)	42 318	(10.0)
Alcohol consumption, *n* (%)												
Never	519	(4.2)	18 122	(4.1)	121	(4.6)	18 520	(4.1)	1628	(5.4)	17 013	(4.0)
Previous drinker	846	(6.9)	14 814	(3.4)	227	(8.6)	15 433	(3.4)	1833	(6.1)	13 827	(3.3)
Current drinker	10 912	(88.9)	407 780	(92.5)	2295	(86.8)	416 397	(92.5)	26 685	(88.5)	392 007	(92.7)
Addition of salt to food, *n* (%)												
Never or rarely	6238	(50.8)	246 184	(55.9)	1325	(50.1)	251 097	(55.8)	15 718	(52.1)	236 704	(56.0)
Sometimes	5180	(42.2)	174 182	(39.5)	1112	(42.1)	178 250	(39.6)	12 463	(41.3)	166 899	(39.5)
Usually or always	859	(7.0)	20 350	(4.6)	206	(7.8)	21 003	(4.7)	1965	(6.5)	19 244	(4.6)
Consumption of oily fish, *n* (%)												
Never	1435	(11.7)	47 516	(10.8)	326	(12.3)	48 625	(10.8)	3505	(11.6)	45 446	(10.7)
Less than once per week	3818	(31.1)	147 304	(33.4)	806	(30.5)	150 316	(33.4)	8999	(29.9)	142 123	(33.6)
At least once per week	7024	(57.2)	245 896	(55.8)	1511	(57.2)	251 409	(55.8)	17 642	(58.5)	235 278	(55.6)
Fruit and vegetable intake score, median (IQR)	1.0	(1.0–2.0)	2.0	(1.0–2.0)	1.0	(1.0–2.0)	2.0	(1.0–2.0)	1.0	(1.0–2.0)	2.0	(1.0–2.0)
Weekly frequency of red or processed meat intake, median (IQR)	0.9	(0.6–1.4)	0.8	(0.5–1.3)	1.0	(0.6–1.4)	0.8	(0.5–1.3)	0.9	(0.6–1.3)	0.8	(0.5–1.3)
Leisure screen time, *n* (%)												
<4 h	4944	(40.3)	227 610	(51.6)	949	(35.9)	231 605	(51.4)	11 496	(38.1)	221 058	(52.3)
≥4 h	7333	(59.7)	213 106	(48.4)	1694	(64.1)	218 745	(48.6)	18 650	(61.9)	201 789	(47.7)
Typical sleep per 24-h period, *n* (%)												
<7 h	3225	(26.3)	107 233	(24.3)	729	(27.6)	109 729	(24.4)	8460	(28.1)	101 998	(24.1)
7–8 h	7449	(60.7)	300 810	(68.3)	1502	(56.8)	306 757	(68.1)	18 107	(60.1)	290 152	(68.6)
>8 h	1603	(13.1)	32 673	(7.4)	412	(15.6)	33 864	(7.5)	3579	(11.9)	30 697	(7.3)
BMI, *n* (%)												
Normal/underweight <25 kg/m^2^	3564	(29.0)	147 232	(33.4)	608	(23.0)	150 188	(33.3)	6072	(20.1)	144 724	(34.2)
Overweight 25–<30 kg/m^2^	5026	(40.9)	187 827	(42.6)	1078	(40.8)	191 775	(42.6)	13 189	(43.8)	179 664	(42.5)
Obese ≥30 kg/m^2^	3687	(30.0)	105 657	(24.0)	957	(36.2)	108 387	(24.1)	10 885	(36.1)	98 459	(23.3)
Current prescription of blood pressure medicine, *n* (%)												
	4422	(36.0)	88 579	(20.1)	1389	(52.6)	91 612	(20.3)	15 464	(51.3)	77 537	(18.3)
Current prescription of cholesterol medicine, *n* (%)												
	3837	(31.3)	73 838	(16.8)	1328	(50.2)	76 347	(17.0)	16 203	(53.7)	61 472	(14.5)
Diagnosis of diabetes or insulin prescription, *n* (%)												
	1563	(12.7)	21 428	(4.9)	602	(22.8)	22 389	(5.0)	4574	(15.2)	18 417	(4.4)
Parental history of CVD, *n* (%)												
	6939	(56.5)	240 351	(54.5)	1592	(60.2)	245 698	(54.6)	19 632	(65.1)	227 658	(53.8)
Parental history of cancer, *n* (%)												
	3989	(32.5)	136 065	(30.9)	791	(29.9)	139 263	(30.9)	9019	(29.9)	131 035	(31.0)
Previous diagnosis (self-report or hospital episode) of CVD or cancer, *n* (%)												
	4692	(38.2)	59 884	(13.6)	1261	(47.7)	63 315	(14.1)	15 707	(52.1)	48 869	(11.6)

a Sample includes all those with prevalent disease and does not exclude early cases. The follow-up time for cardiovascular disease incidence was median of 6.1 years.

**Figure 1.**
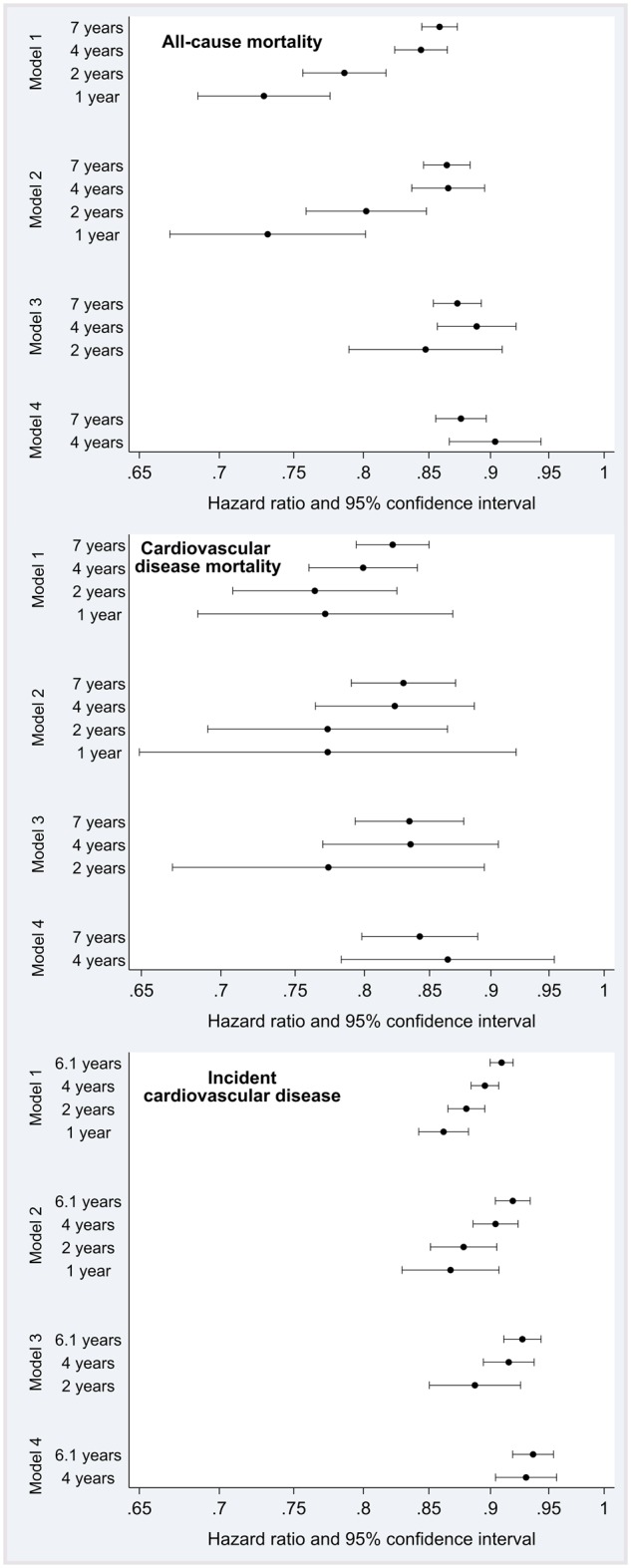
Prospective associations between physical activity and health outcomes by modelling approach and follow-up time, in the UK Biobank study (2006–2010 to 2015–2016). 1, 2 and 4 years of follow-up time are cut-off values; 6.1 and 7 years are median values. The log-hazard ratios estimate the increase in risk of the outcome for an increase of 1 standard deviation in the log(min of MVPA + 1). All analyses were adjusted for covariates listed in the text. Model 1: also adjusted for prevalent disease (CVD and cancer); Model 2: excluded those with prevalent disease; Model 3: Model 2 + excluded cases occurring in first year of follow-up; Model 4: Model 2 + excluded cases occurring in first 2 years of follow-up.

Figure 1 and Table 2 show the hazard ratios (HRs) for a one standard deviation difference in the transformed physical activity variable, for each combination of outcome, model and follow-up time cut-off. With a few exceptions, for any given model the strength of the association decreased as follow-up time increased (Table 2). The greatest difference was seen for all-cause mortality. For example, for Model 1, the hazard ratio was 0.86 (0.84–0.87) after 7 years of follow-up compared with 0.73 (0.69–0.78) after 1 year, i.e. a 2-fold difference in magnitude (log hazard ratios −0.32 vs −0.15). The equivalent estimates for CVD mortality and incidence were approximately 30–70% higher. There were some exceptions to this in the models that excluded those with early events (Models 3 and 4) after the longer follow-up times (4 and 7 years), but the percentage differences were of smaller magnitudes (<25%).

**Table 2.** Prospective associations between physical activity and health outcomes by modelling approach and follow-up time, in the UK Biobank study (2006–2010 to 2015–2016)

Model (*n*)	Follow-up time^a^	Cases	Person-years	Hazard ratio (95% CI)	% Difference in log hazard ratio from longest follow up time
All- cause mortality					
Model 1 (452 993)	7 years	12 277	3 130 875	0.86 (0.84–0.87)	–
	4 years	5509	1 802 723	0.84 (0.82–0.86)	11
	2 years	2102	904 227	0.79 (0.76–0.82)	58
	1 year	806	452 658	0.73 (0.69–0.78)	107
Model 2 (388 417)	7 years	7585	2 695 399	0.86 (0.85–0.88)	–
	4 years	3093	1 548 749	0.87 (0.84–0.90)	−1
	2 years	1062	775 980	0.80 (0.76–0.85)	51
	1 year	381	388 262	0.73 (0.67–0.80)	114
Model 3 (388 036)	7 years	7204	2 695 173	0.87 (0.85–0.89)	–
	4 years	2712	1 548 523	0.89 (0.86–0.92)	−13
	2 years	681	775 754	0.85 (0.79–0.91)	22
Model 4 (387 355)	7 years	6523	2 694 129	0.88 (0.86–0.90)	–
	4 years	2031	1 547 479	0.90 (0.87–0.94)	−24
Cardiovascular disease mortality					
Model 1 (452 993)	7 years	2643	3 130 875	0.82 (0.79–0.85)	–
	4 years	1177	1 802 723	0.80 (0.76–0.84)	14
	2 years	505	904 227	0.76 (0.71–0.82)	37
	1 year	210	452 658	0.77 (0.69–0.87)	32
Model 2 (388 417)	7 years	1382	2 695 399	0.83 (0.79–0.87)	–
	4 years	601	1 548 749	0.82 (0.76–0.89)	4
	2 years	249	775 980	0.77 (0.69–0.86)	38
	1 year	103	388 262	0.77 (0.65–0.92)	38
Model 3 (388 314)	7 years	1279	2 695 342	0.83 (0.79–0.88)	–
	4 years	498	1 548 692	0.84 (0.77–0.91)	−1
	2 years	146	775 923	0.77 (0.67–0.89)	42
Model 4 (388 168)	7 years	1133	2 695 128	0.84 (0.80–0.89)	–
	4 years	352	1 548 478	0.86 (0.78–0.95)	−15
Incident cardiovascular disease					
Model 1 (452 993)	6.1 years	30 146	2 709 543	0.91 (0.90–0.92)	–
	4 years	20 479	1 762 204	0.90 (0.88–0.91)	16
	2 years	11 124	893 077	0.88 (0.87–0.90)	34
	1 year	6005	449 640	0.86 (0.84–0.88)	57
Model 2 (388 417)	6.1 years	14 439	2 373 103	0.92 (0.90–0.93)	–
	4 years	8524	1 534 185	0.90 (0.89–0.92)	19
	2 years	3802	772 609	0.88 (0.85–0.91)	54
	1 year	1781	387 470	0.87 (0.83–0.91)	68
Model 3 (386 636)	6.1 years	12 658	2 372 164	0.93 (0.91–0.94)	–
	4 years	6743	1 533 246	0.92 (0.89–0.94)	17
	2 years	2021	771 669	0.89 (0.85–0.93)	58
Model 4 (384 615)	6.1 years	10 637	2 369 116	0.94 (0.92–0.95)	–
	4 years	4722	1 530 198	0.93 (0.90–0.96)	10

a 1, 2, and 4 years of follow-up time are cut-off values; 6.1 and 7 years are median values.

The log-hazard ratios estimate the increase in risk of the outcome for an increase of 1 standard deviation in the log(min of MVPA + 1). All analyses were adjusted for age, sex, BMI, smoking status, education, deprivation, sleep, leisure screen time, salt intake, oily fish intake, fruit and vegetable intake, processed/red meat intake, blood pressure medication, cholesterol medication, diabetes and/or insulin medication, parental history of cardiovascular disease and parental history of cancer. The baseline hazards were stratified by assessment centre, ethnicity, alcohol intake, employment/active commuting/manual work status. Model 1: adjusted for prevalent disease (cardiovascular disease and cancer); Model 2: excluded those with prevalent disease; Model 3: Model 2 + excluded cases occurring in first year of follow-up; Model 4: Model 2 + excluded cases occurring in first 2 years of follow-up.

**Figure 2.**
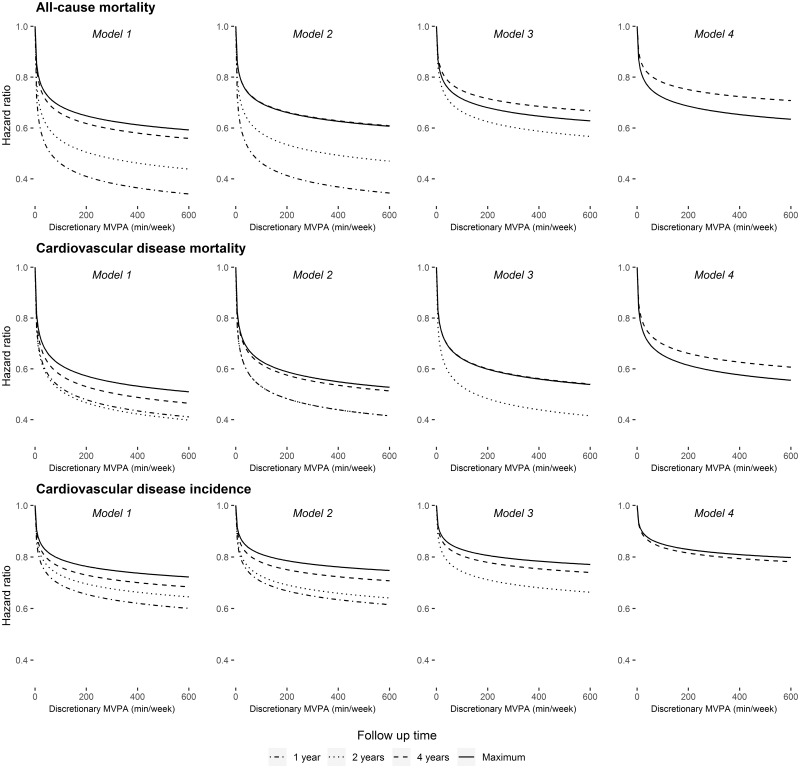
Dose–response relationships between physical activity and health outcomes at 1, 2, 4 and 7 years of follow-up (6.1 years for CVD incidence) using four modelling approaches in the UK Biobank study (2006–2010 to 2015–2016). All analyses were adjusted for covariates listed in the text. Model 1: also adjusted for prevalent disease (CVD and cancer); Model 2: excluded those with prevalent disease; Model 3: Model 2 + excluded cases occurring in first year of follow-up; Model 4: Model 2 + excluded cases occurring in first 2 years of follow-up.

Supplementary Table 1 and Figure 2, available as Supplementary data at *IJE*, online display these same estimates, re-arranged to allow direct comparison between models for the same follow-up time cut-off. With the maximum available follow-up time, the estimates from Model 4 (excluding those with prevalent disease and those experiencing the outcome within the first 2 years of follow-up) were attenuated compared with those from Model 1 (adjustment for prevalent disease only). The relative differences between models were greatest in analyses using 4-years of follow-up. The relative differences between models were greatest for incident CVD, although the absolute differences were similar for the other outcomes.

To assist with interpretation, Figure 2 shows the HRs for the different follow-up times by model across the range of 0–600 min/week of discretionary MVPA. Supplementary Figure 3, available as Supplementary data at *IJE* online, displays these estimates re-arranged to facilitate comparisons between models across the different follow-up times.

The coefficients from regression of standardized physical activity at follow-up on baseline were 0.2 (SE 0.020) for those who undertook the first re-visit <3 years after baseline, 0.49 (SE 0.007) for whole first re-visit sample (median 4.4 years after baseline) and 0.39 (SE 0.006) second re-visit (median 7.6 years after baseline).

The results of the sensitivity analyses without adjustment for covariates potentially on the causal pathway were almost identical to the main analyses (data not shown).

## Discussion

With increasing availability of large datasets (e.g. biobanks), researchers face important decisions alongside the unique opportunities offered by these resources. This study is the first to quantify the combined impact of choice of follow-up time cut-off and method to address reverse causality bias on estimates of association between self-reported physical activity and these outcomes. We found that choice of cut-off for follow-up time within a range of 1–7 years can strongly influence the magnitude of the prospective association. When all-cause mortality was the outcome, the log HRs based on a maximum follow-up of 1 year were over double the magnitude of those obtained using a median 7-year follow-up. There may be a number of explanations for the attenuation of the association with increasing follow-up time, further to the reduced influence of reverse causality bias. For example, we would expect individual variation in physical activity levels, resulting in the baseline exposure measurement not perfectly reflecting exposure over the entire follow-up period. As Skogstad *et al*. showed, some health benefits of physical activity may not persist if levels are not maintained: after an 8 week physical activity intervention, both physical activity levels and various biomarkers of CVD risk returned to baseline levels 15 months later.^20^ Therefore, one should expect some attenuation as follow-up time increases. However, since the magnitude of the regression dilution over the 3–7 years after baseline were estimated to be fairly similar in this study, it is unlikely that changes in physical activity behaviour fully explain these differences. Excluding individuals with prevalent disease attenuated the estimated associations compared with adjusting for it, as did excluding early incident cases to address the issue of undiagnosed underlying disease impacting physical activity levels. However, the impact of different analytical approaches for dealing with reverse causality was smaller as follow-up time increased. Our results are comparable with the findings of Andersen *et al*. who observed attenuation in the associations of physical activity levels with all-cause mortality over a 10-year period in Danish adults.^9^ The HR comparing the risk of all-cause mortality for least active individuals with that of the highly active decreased from 2.6 to 1.9 when follow-up lengthened from 2 to 10 years, representing ∼30% difference on the log scale. The most comparable result in the present study was the 55% attenuation of the Model 2 estimates between 2- and 7-year follow-up. Different exposure measures and modelling choices, sample sizes and event rates, and sample characteristics are likely to influence the level of attenuation.^21^

One other study investigated differences in the association between physical activity and health outcomes by different cut-offs for follow-up time up to 7 years. De Bruijn *et al*. found attenuation in the association between physical activity and dementia over a 2–6 year period, to the extent that there was no evidence of an association after 5 years.^22^ In response to this finding, the authors discussed the need to consider the interplay between physical activity and the disease pathway when choosing the follow-up time. However for this outcome, there are further complexities to consider, as the disease state (even at a pre-diagnostic stage) may also negatively impact on the subjective recall of physical activity. Therefore, despite their rigorous screening methods at baseline, correlated measurement error may also explain the associations.

A major strength of the current work is its relevance to the decisions faced by researchers today. We used a large sample of middle-aged adults where event rates may in principle be high enough to undertake association analyses soon after baseline. By quantifying the difference in the estimates, we have assisted those who meta-analyse results from studies with different follow-up periods in the <10 year range. Our results are particularly timely as the follow-up time for the UK Biobank subsample with accelerometry measures (undertaken ∼3 years after baseline interview^23^) will soon be sufficient for prospective analyses. However, it remains unclear whether our findings would also apply to a situation where physical activity is measured objectively. This will be important to investigate to aid interpretation of the results of two recent studies^8^^,^^24^ reporting on the associations between accelerometer-derived physical activity and mortality after 1–2 years of follow-up. Accelerometer-derived metrics may be more precise at differentiating between levels of physical activity than self-report methods,^25^ which may change the strength of the association with health outcomes and cross-sectionally with underlying disease. If so, it is possible that there may be a different pattern of variation in the estimates based on different lengths of follow-up and ways of accounting for reverse causality. This study is also the first to present associations of this particular physical activity exposure summary measure and all-cause and CVD mortality and CVD incidence in the UK Biobank sample. Previous work has either reported associations by domain^26^ or has used summary measures from the International Physical Activity Questionnaire Short Form.^7^

There are also some limitations of our work. The UK Biobank sample is non-representative of the general population (5.5% response rate) and has been shown to be healthier than the UK population.^27^ This may affect the generalizability of the results. Potentially, the associations we observe in this study would likely be greater still in older or less healthy populations who have an increased prevalence of underlying disease, or for whom the timeline of disease progression may be different. Therefore, similar work in other population samples is needed. We have also only investigated one behavioural exposure; the pattern of associations may also be present for other exposures, for example specific sedentary behaviours or food intake. Lastly, ∼30% of the UK Biobank participants have been identified as having at least one third-degree or closer relative in the study. We have not accounted for this relatedness in our analyses, thus the certainty of individual HRs may be slightly overestimated. However, this should not impact on our main conclusions, as we focus here on the relative difference between models rather than absolute associations.

In conclusion, we have shown important differences in the associations between self-reported physical activity and all-cause mortality and CVD outcomes as follow-up time increases over a 7 year period. We have also shown that analytical approaches to account for reverse causality can affect estimates, particularly with shorter follow-up times. The expected time course of disease progression is critical to these decisions, and, as such, just because analyses can be undertaken, it does not mean that they should be.

## Funding

This work was supported by the Medical Research Council [grant numbers MC_UU_12015/1 and MC_UU_12015/3] (T.S., K.W., S.J.S., P.C.D., N.W., S.B.) and a National Health and Medical Research Council of Australia research fellowship [No. 1142685] (P.C.D.).

## Supplementary Material

dyz212_Supplementary_Materials
