# Supplementary material for: Impact of follow-up time and analytical approaches to account for reverse causality on the association between physical activity and health outcomes in UK Biobank
Source: Int J Epidemiol. 2019 Oct 25;49(1):162–72. doi: 10.1093/ije/dyz212 (PMC7124507; doi:10.1093/ije/dyz212)
Supplement: dyz212_Supplementary_Materials [file dyz212_supplementary_materials.zip › dyz212-suppl_data/Supplementary Figure 1.docx]

Supplementary Figure 1.

Hazard ratios from cubic spline Cox regressions for the transformed physical activity variable to assess log-linearity.


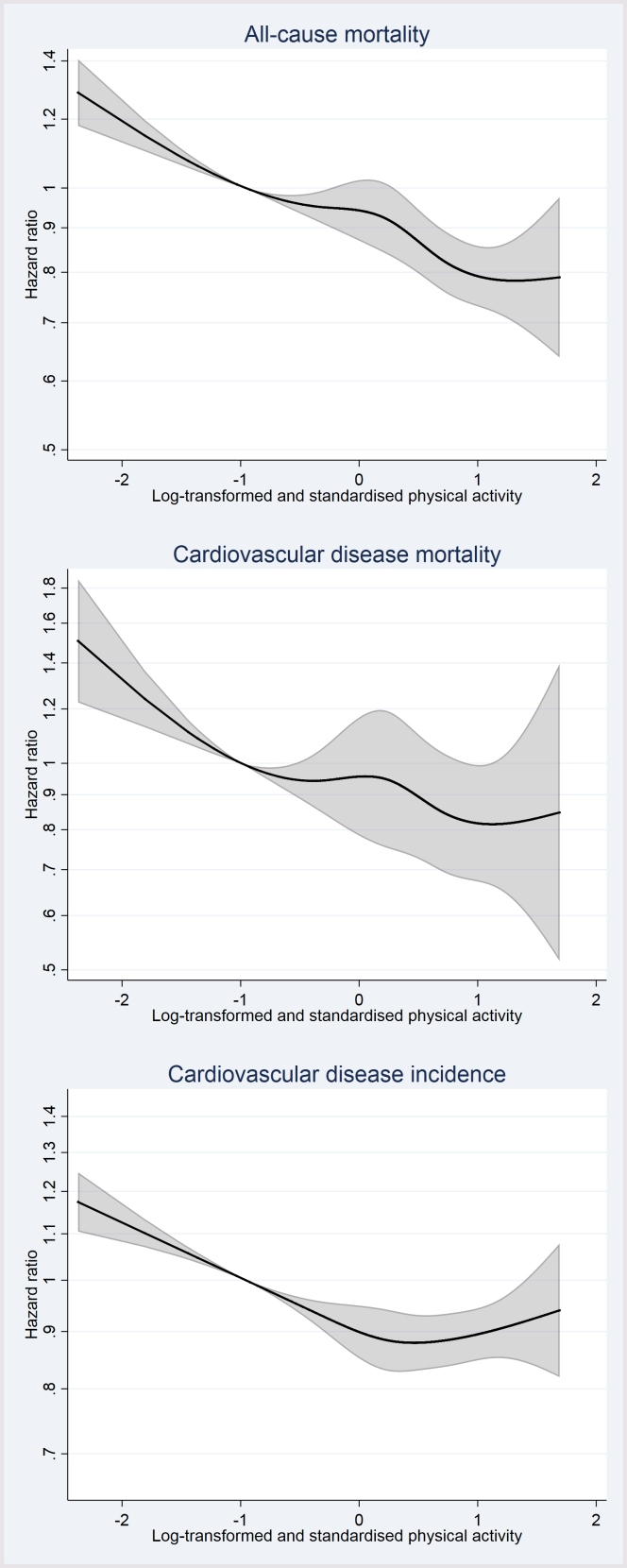


This figure shows that the relationships between the exposure variable (log-transformed and standardised minutes of physical activity per week, described in the main manuscript) and the outcomes of all-cause mortality, cardiovascular disease mortality and incidence are approximately log-linear. The assumption of a log-linear relationship underpins our decision to include the transformed variable as a continuous variable in the models. We used cubic spline regressions with 4 evenly-spaced knots across the exposure distribution and a reference value of -1 on the transformed variable to produce these plots.
