# Supplementary material for: Impact of follow-up time and analytical approaches to account for reverse causality on the association between physical activity and health outcomes in UK Biobank
Source: Int J Epidemiol. 2019 Oct 25;49(1):162–72. doi: 10.1093/ije/dyz212 (PMC7124507; doi:10.1093/ije/dyz212)
Supplement: dyz212_Supplementary_Materials [file dyz212_supplementary_materials.zip › dyz212-suppl_data/Supplementary Table 1.docx]

Supplementary Table 1. Prospective associations between physical activity and health outcomes by follow-up time and modelling approach, in the UK Biobank study (2006-2010 to 2015-2016).

| Follow-up time^a^ | Model | | Hazard ratio | | % difference in log hazard ratio from Model 1 | |
| --- | --- | --- | --- | --- | --- | --- |
| All- cause mortality |  | |  | |  | |
| 7 years | Model 1 | | 0.86 (0.84-0.87) | | - | |
|  | Model 2 | | 0.86 (0.85-0.88) | | -4 | |
|  | Model 3 | | 0.87 (0.85-0.89) | | -11 | |
|  | Model 4 | | 0.88 (0.86-0.90) | | -13 | |
| 4 years | Model 1 | | 0.84 (0.82-0.86) | | - | |
|  | Model 2 | | 0.87 (0.84-0.90) | | -15 | |
|  | Model 3 | | 0.89 (0.86-0.92) | | -30 | |
|  | Model 4 | | 0.90 (0.87-0.94) | | -41 | |
| 2 years | Model 1 | | 0.79 (0.76-0.82) | | - | |
|  | Model 2 | | 0.80 (0.76-0.85) | | -8 | |
|  | Model 3 | | 0.85 (0.79-0.91) | | -31 | |
| 1 year | Model 1 | | 0.73 (0.69-0.78) | | - | |
|  | Model 2 | | 0.73 (0.67-0.80) | | -1 | |
| Cardiovascular disease mortality | | | |  | |  |
| 7 years | Model 1 | | 0.82 (0.79-0.85) | | - | |
|  | Model 2 | | 0.83 (0.79-0.87) | | -5 | |
|  | Model 3 | | 0.83 (0.79-0.88) | | -8 | |
|  | Model 4 | | 0.84 (0.80-0.89) | | -13 | |
| 4 years | Model 1 | | 0.80 (0.76-0.84) | | - | |
|  | Model 2 | | 0.82 (0.76-0.89) | | -13 | |
|  | Model 3 | | 0.84 (0.77-0.91) | | -20 | |
|  | Model 4 | | 0.86 (0.78-0.95) | | -35 | |
| 2 years | Model 1 | | 0.76 (0.71-0.82) | | - | |
|  | Model 2 | | 0.77 (0.69-0.86) | | -4 | |
|  | Model 3 | | 0.77 (0.67-0.89) | | -5 | |
| 1 year | Model 1 | | 0.77 (0.69-0.87) | | - | |
|  | Model 2 | | 0.77 (0.65-0.92) | | -1 | |
| Incident cardiovascular disease | |  |  |  | |  |
| 6.1 years | Model 1 | | 0.91 (0.90-0.92) | | - | |
|  | Model 2 | | 0.92 (0.90-0.93) | | -11 | |
|  | Model 3 | | 0.93 (0.91-0.94) | | -20 | |
|  | Model 4 | | 0.94 (0.92-0.95) | | -31 | |
| 4 years | Model 1 | | 0.90 (0.88-0.91) | | - | |
|  | Model 2 | | 0.90 (0.89-0.92) | | -9 | |
|  | Model 3 | | 0.92 (0.89-0.94) | | -20 | |
|  | Model 4 | | 0.93 (0.90-0.96) | | -35 | |
| 2 years | Model 1 | | 0.88 (0.87-0.90) | | - | |
|  | Model 2 | | 0.88 (0.85-0.91) | | 2 | |
|  | Model 3 | | 0.89 (0.85-0.93) | | -6 | |
| 1 year | Model 1 | | 0.86 (0.84-0.88) | | - | |
|  | Model 2 | | 0.87 (0.83-0.91) | | -4 | |

^a^1, 2, and 4 years of follow-up time are cut-off values; 6.1 and 7 years are median values.

The log-hazard ratios estimate the increase in risk of the outcome for an increase of 1 standard deviation in the log(mins of MVPA+1). All analyses were adjusted for age, sex, BMI, smoking status, education, deprivation, sleep, leisure screen time, salt intake, oily fish intake, fruit and vegetable intake, processed/red meat intake, blood pressure medication, cholesterol medication, diabetes and/or insulin medication, parental history of cardiovascular disease, and parental history of cancer. The baseline hazards were stratified by assessment centre, ethnicity, alcohol intake, employment/active commuting/manual work status. Model 1: adjusted for prevalent disease (cardiovascular disease and cancer); Model 2: excluded those with prevalent disease; Model 3: Model 2 + excluded cases occurring in first year of follow-up; Model 4: Model 2 + excluded cases occurring in first two years of follow-up.
