# Supplementary figures and images for: Impact of follow-up time and analytical approaches to account for reverse causality on the association between physical activity and health outcomes in UK Biobank
Source: Int J Epidemiol. 2019 Oct 25;49(1):162–72. doi: 10.1093/ije/dyz212 (PMC7124507; doi:10.1093/ije/dyz212)

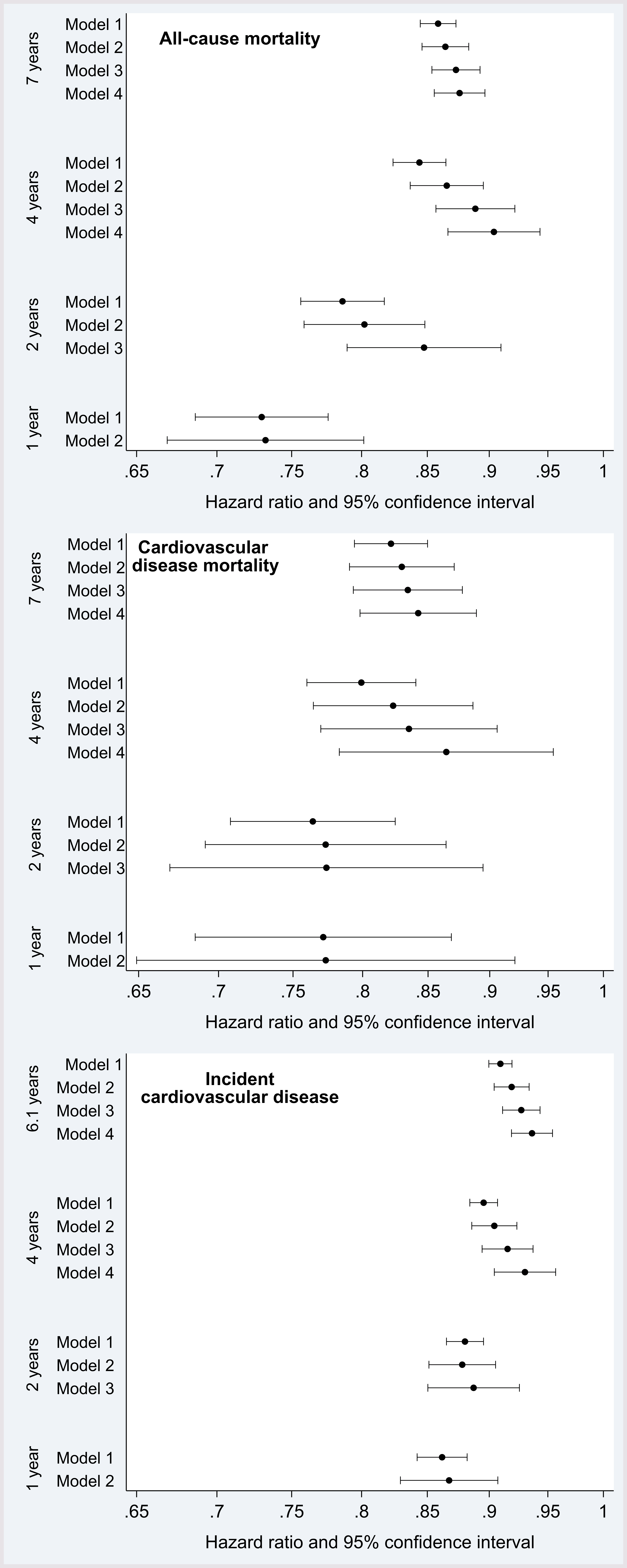

Supplement: dyz212_Supplementary_Materials [file dyz212_supplementary_materials.zip › dyz212-suppl_data/Supplementary Figure 1.png]

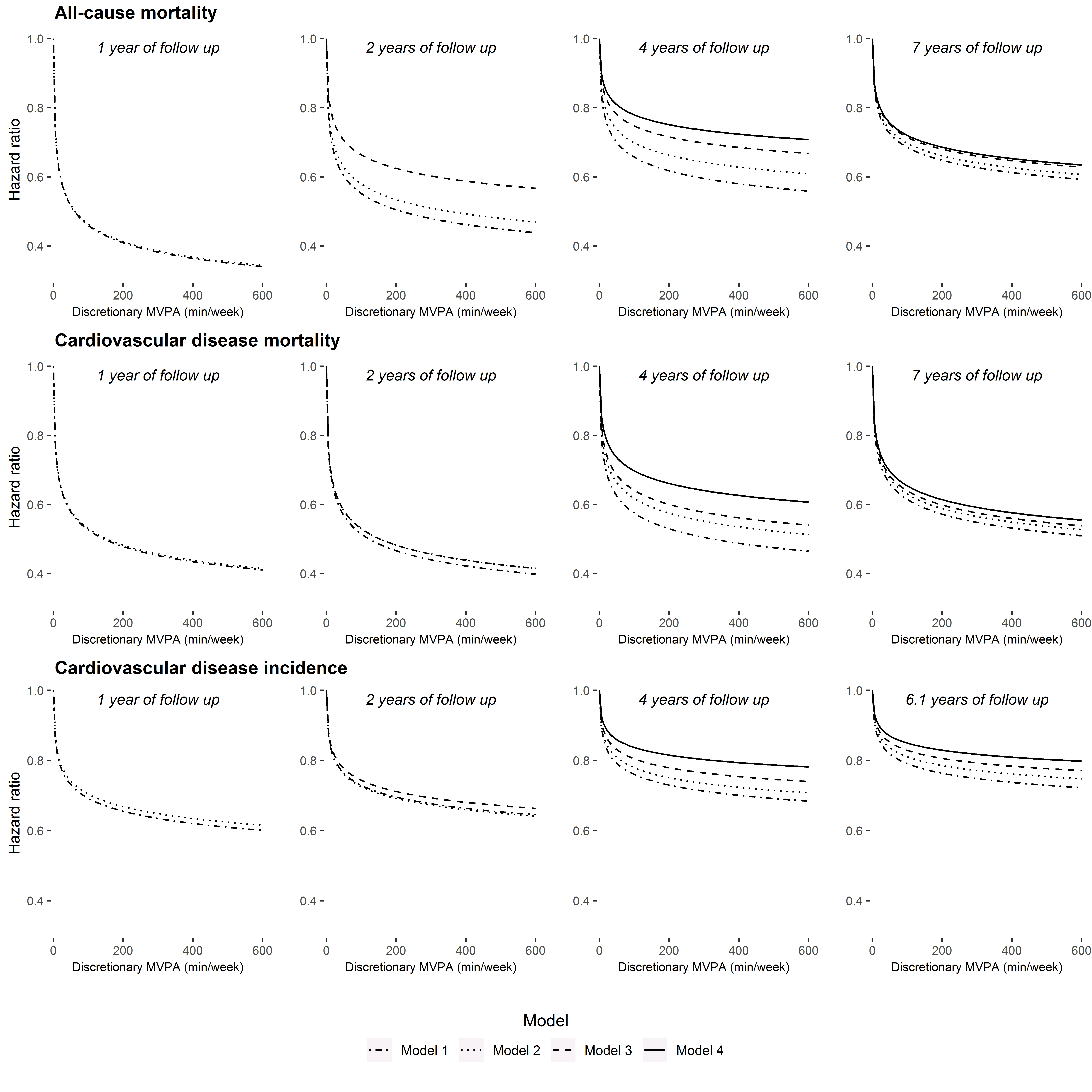

Supplement: dyz212_Supplementary_Materials [file dyz212_supplementary_materials.zip › dyz212-suppl_data/Supplementary Figure 2.png]
